# Imipramine Protects against Bone Loss by Inhibition of Osteoblast-Derived Microvesicles

**DOI:** 10.3390/ijms18051013

**Published:** 2017-05-08

**Authors:** Lili Deng, Ying Peng, Yuhai Jiang, Yu Wu, Yuedi Ding, Yaping Wang, Dong Xu, Qiang Fu

**Affiliations:** 1 Key Laboratory of Nuclear Medicine, Ministry of Health, Jiangsu Institute of Nuclear Medicine, Wuxi 214063, Jiangsu, China; pengying@jsinm.org (Y.P.); dingyuedi@jsinm.org (Y.D.); xudong@jsinm.org (D.X.); 2 Wuxi Second People’s Hospital of Nanjing Medical University, Wuxi 214123, Jiangsu, China; jyh_wj@sina.com (Y.J.); ypwnag0301@126.com (Y.W.); 3 Wuxi Medical School, Jiangnan University, Wux i214122, Jiangsu, China; wuyu@jiangnan.edu.cn

**Keywords:** imipramine, microvesicles, cellular communication, bone loss

## Abstract

The maintenance of bone homeostasis is largely dependent upon cellular communication between osteoclasts and osteoblasts. Microvesicles (MVs) represent a novel mechanism for osteoblasts and osteoclasts communication, as has been demonstrated in our previous study. Sphingomyelinases catalyze the hydrolysis of sphingomyelin, which leads to increased membrane fluidity and facilitates MV generation. This effect can be inhibited by imipramine, an inhibitor of acid sphingomyelinase (ASM), which is also known as a member of tricyclic antidepressants (TCAs). A recent study has reported that in vitro treatment of imipramine blocked MVs release from glial cells. However, whether imipramine has this effect on osteoblast-derived MVs and whether it is involved in MV generation in vivo is unclear. Here, our investigations found that imipramine slightly reduced the expression of osteoblast differentiation of related genes, but did not impact parathyroid hormone (PTH) regulation for these genes and also did not affect receptor activator of nuclear factor-κB ligand (RANKL)-mediated osteoclast formation; however, imipramine treatment blocked MVs released from osteoblasts and inhibited MV-induced osteoclast formation. In vivo, mice administrated with imipramine were protected from ovariectomy-induced bone loss as evaluated by various bone structural parameters and serum levels of biochemical markers. Our results suggest that inhibiting the production of MVs containing RANKL in vivo is very important for preventing bone loss.

## 1. Introduction

Bone remodeling is the predominant metabolic process regulating bone structure and function during adult life. Osteoclastic bone resorption coupled with bone formation helps to maintain skeletal integrity and mineral homeostasis, which is tightly controlled and largely dependent upon cellular communication between osteoclasts and osteoblasts.

Classical intercellular communication can be mediated through direct cell–cell contact or through the transfer of secreted molecules. Recent studies have suggested that intercellular transfer of extracellular vesicles, which carry a diverse array of signaling molecules including nucleic acids, proteins, and lipids, is another mechanism for important cell–cell communication [1,2,3,4]. Microvesicles (MVs) and exosomes are two classes of extracellular vesicles released by cells into the extracellular space via a distinct mechanism. MVs typically range in size from 100 to about 1000 nm in diameter and are formed by the direct budding of the plasma membrane into the extracellular space, while exosomes are significantly smaller than MVs, averaging 30 to 100 nm in size, and are produced within the cell and released through exocytosis events. Both MVs and exosomes have an aqueous, cargo-containing core surrounded by a roughly spherical bilayer membrane, which protects the contents from extracellular degradation and allows for the more precise targeting of the contents [5]. MVs have prominent exposure of phosphatidylserine (PS) on their outer surfaces [6]. Sphingomyelin (SM), a phospholipid abundant in the outer leaflet of the plasma membrane, has a high affinity for cholesterol and both lipids are major determinants of the membrane fluidity and structural integrity of the plasma membrane [7]. The enzyme family of sphingomyelinases (SMases) catalyzes the hydrolysis of SM, leading to an increased efflux of cholesterol and membrane fluidity [8,9], thus inducing membrane destabilization and facilitating membrane blebbing and MV shedding [10,11].

Imipramine (IMI) is a member of the tricyclic antidepressants (TCAs), which were discovered to inhibit acid sphingomyelinase (ASM) [12]. ASM is a downstream molecule of ionotropic ATP receptor P2X7 (P2X7R) signaling, and P2X7R simulation leads to the activation of ASM with rapid SM hydrolysis and MV shedding. A previous study has shown that the in vitro treatment of imipramine blocked P2X7R-dependent and independent MV generation from glial cells by inhibiting ASM activation [13]. In our previous study, we demonstrated that MVs shed from osteoblasts contain the cell-specific surface membrane receptor, RANKL (receptor activator of nuclear factor-κB ligand) protein, and can transfer to the surface of osteoclast precursors through receptor-ligand interaction (RANKL–RANK), leading to the activation of the RANKL–RANK signaling pathway to facilitate osteoclast formation [14].

To further understand the importance of MVs in the regulation of bone metabolism, in this study, we investigated whether in vivo administration of imipramine to block the production of MVs would reduce bone loss caused by estrogen deficiency. As very few articles reported the direct effect of imipramine on bone, before in vivo study, we firstly observed the influence of imipramine on osteoblast genes expression and osteoclast formation in vitro.

## 2. Results

### 2.1. Imipramine Blocks MV Generation from Osteoblasts

**Figure 1:**
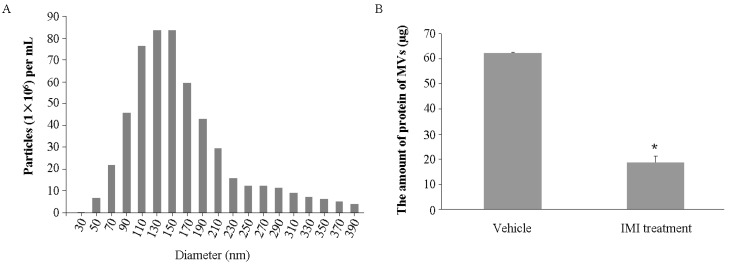
Characterization of osteoblast-derived microvesicles (MVs). (**A**) The size distribution and total number of microvesicles from osteoblast were measured by Nanoparticle Tracking Analysis (NanoSight); (**B**) The total amount of protein of MVs shed from UAMS-32P cells with or without imipramine treatment (obtained from 4 × 10^7^ cells). Each bar represents the mean ± standard deviation (*n* = 3). * *p* < 0.05 by Student’s *t* test. Vehicle: without imipramine treatment; IMI: with imipramine treatment.

As known, MVs release from almost all cell types. UAMS-32P cells, the murine stromal/osteoblastic cell line, produced abundant MVs in the culture medium. Based on nanoparticle tracking analysis (NTA), we showed that a typical total number of MVs shed from 4 × 10^7^ UAMS-32P cells was about 5.65 × 10^8^ particles, while the number was decreased to 2.54 × 10^8^ particles with imipramine treatment. The diameters of these MVs were from 50 to 390 nm; most of them were between 90 to 190 nm and the mean size was about 180 nm (Figure 1A). As expected, the total amount of protein of MVs released from the cells treated with imipramine was significantly decreased compared with the vehicle group (Figure 1B), indicating that imipramine treatment indeed blocked MV generation.

### 2.2. Imipramine Slightly Reduces Osteoblast Differentiation Gene Expression, but Does Not Affect Parathyroid Hormone Regulation

**Figure 2:**
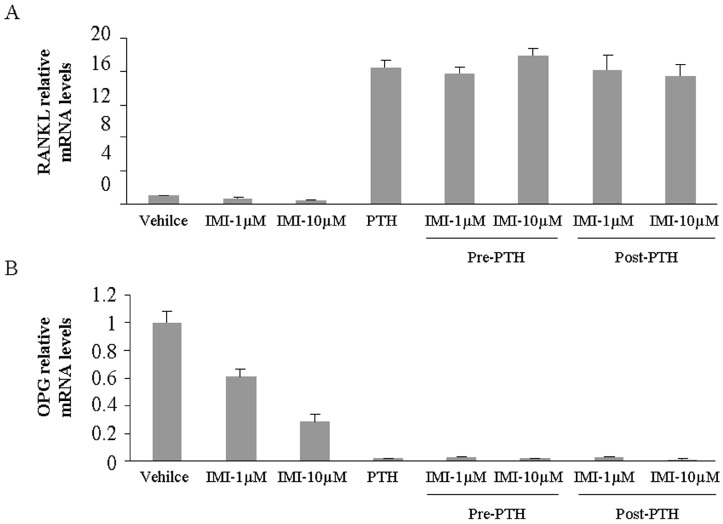

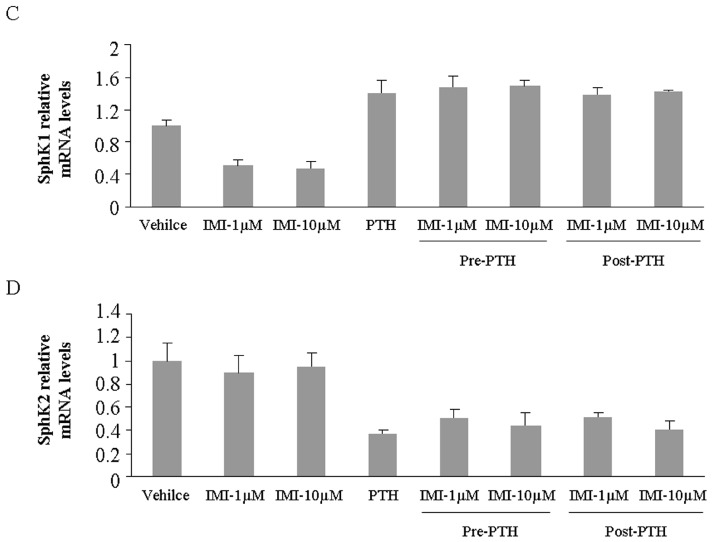
Imipramine regulates genes expression in osteoblasts. (**A**) mRNA expression for *RANKL*; (**B**) mRNA expression for *OPG*; (**C**) mRNA expression for *SphK1*; (**D**) mRNA expression for *SphK2*. Each bar represents the mean ± standard deviation (*n* = 3). Vehicle: treatment with PBS; IMI: treatment with imipramine; PTH: treatment with PTH; Pre-PTH: one hour pre-treatment with PTH and then an added imipramine treatment; Post-PTH: one hour imipramine pre-treatment and then an added PTH treatment.

RANKL and OPG (osteoprotegerin) are produced by osteoblastic cells and have important roles in regulating osteoclast formation. Parathyroid hormone (PTH) stimulates *RANKL* up-regulation and inhibits *OPG* down-regulation in primary cultures of stromal/osteoblastic cells and osteoblastic cell line [15]. To investigate the effect of imipramine on bone metabolism, we measured these two gene expression levels in UAMS-32P cells after imipramine treatment. The results showed that imipramine alone slightly reduced the basal expression level of *RANKL* and *OPG*, but the regulation of PTH for these two gene expressions was not affected by imipramine pre- or post-treatment (Figure 2A,B). Similarly, imipramine also did not change the regulation of PTH for other osteoblast differentiation genes, such as *BMP2*, *Runx2*, and *Osterix* in UAMS-32P cells (Figure S1).

Sphingosine kinase (SphK) is a lipid kinase that phosphorylates sphingosine to generate sphingosine 1-phosphate (S1P), an important lipid mediator. S1P plays an important role in osteoblast migration, survival and osteoclastogenesis regulation [16,17]. Therefore, we also investigated the gene expression of *SphK1* and *SphK2* in UAMS-32P cells treated with imipramine. The results showed that imipramine alone lightly down-regulated the basal level of *SphK1* gene expression but did not impact *SphK2* gene expression level. PTH up-regulated *SphK1* and down-regulated *SphK2* gene expression in osteoblasts, however, the regulation was not affected by imipramine pre- or post-treatment (Figure 2C,D).

### 2.3. Imipramine Does Not Affect RANKL-Mediated Osteoclast Formation

**Figure 3:**
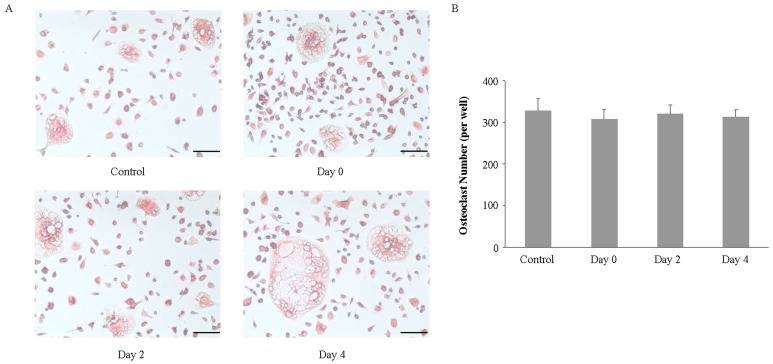
Imipramine did not inhibit RANKL-mediated osteoclast formation. (**A**) Non-adherent bone marrow cells were induced by RANKL plus M-CSF for 6 days to generate mature osteoclasts in the absence of imipramine (control group) or in the presence of imipramine (10 µmol/L) at the beginning of the cell culture (Day 0 group), 2 days after the cell culture (Day 2 group), or 4 days after the cell culture (Day 4 group). Cells were fixed and stained for TRAP; (**B**) The numbers of TRAP staining positive cells were calculated. Each bar represents the mean ± standard deviation in a 24-wellplate (*n* = 3). Bar, 100 µm.

**Figure 4:**
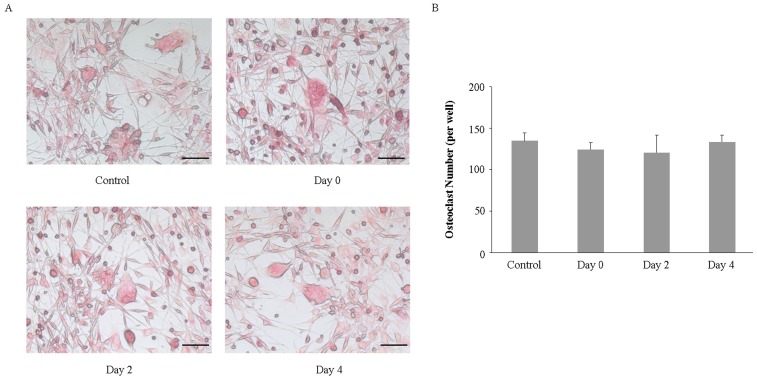
Imipramine did not inhibit osteoclast formation. (**A**) Non-adherent bone marrow cells were co-cultured with UAMS-32P cells in 0.1 µmol/L PTH for 6 days to generate mature osteoclasts in the absence of imipramine (control group) or in the presence of imipramine (10 µmol/L) at the beginning of the cell culture (Day 0 group), 2 days after the cell culture (Day 2 group), or 4 days after the cell culture (Day 4 group). Cells were fixed and stained for TRAP; (**B**) The numbers of TRAP staining positive cells were calculated. Each bar represents the mean±standard deviation in a 24-wellplate (*n* = 3). Bar, 100 µm.

Next, we investigated whether imipramine affected RANKL-mediated osteoclast formation. Non-adherent bone marrow cells were induced to differentiate to osteoclasts in the presence of RANKL plus M-CSF. Imipramine was added to the culture medium at the beginning of the cell culture, or 2 or 4 days after the cell culture. Osteoclast-specific marker TRAP staining showed that imipramine, no matter when it was added to the culture medium during the cell culture, did not inhibit non-adherent bone marrow cells from generating TRAP-positive osteoclasts (Figure 3). Likewise, imipramine also did not interfere in RANKL-mediated osteoclast formation in non-adherent bone marrow cells co-cultured with UAMS-32P cells after PTH stimulation (Figure 4).

### 2.4. Imipramine Affects MV-Induced Osteoclast Formation

**Figure 5:**
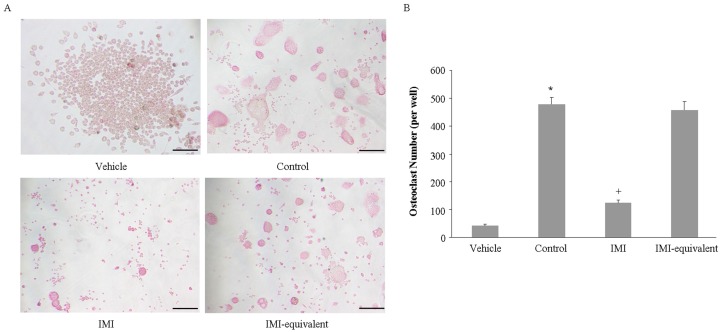
Imipramine affects MV-induced osteoclast formation. (**A**) RAW264.7 cells and MVs shed from PTH-treated UAMS-32P cells with or without imipramine treatment were co-cultured. Cells were fixed and stained for TRAP; (**B**) The numbers of TRAP staining positive cells. Each bar represents the mean±standard deviation in a 24-wellplate (*n* = 3). * *p* < 0.05 versus vehicle group; + *p* < 0.05 versus control group. Vehicle: RAW264.7 cells cultured without MVs; Control: co-culture with MVs shed from PTH-treated osteoblasts without imipramine treatment; IMI: co-culture with MVs shed from PTH-treated osteoblasts with imipramine treatment; IMI-equivalent: co-culture withthe same amount of MVs from the IMI group as the control group. Bar, 100 µm.

Although we showed that imipramine significantly blocked MV generation, we also observed that imipramine did not affect RANKL-mediated osteoclast formation. It is not clear whether imipramine changes the cargo of MVs in order to affect MVs function. To explore this question, RAW264.7 cells, a macrophage cell line which can differentiate to osteoclast after RANKL stimulation, were co-cultured for 6 days with the same volume of MVs, which were collected from 4 × 10^7^ PTH-stimulated UAMS-32P cells in the absence or presence of imipramine. TRAP staining showed that the number of TRAP-positive multinuclear cells induced by the MVs collected from imipramine-treated osteoblasts was remarkably lower than the number observed in cells without imipramine treatment (Figure 5). If equivalent MVs were added into the co-culture medium, by increasing the volume of MV suspension collected from the imipramine-treated osteoblasts, the TRAP-positive multinuclear cells generated in the co-culture system did not show a statistical significance between the MVs shed from the imipramine-treated or non-treated osteoblasts. The results indicated that imipramine inhibited MV-induced osteoclast formation by blocking MV generation, thereby reducing the signaling required for osteoclast differentiation and activation.

### 2.5. Imipramine Protects against Ovariectomy-Induced Bone Loss

Whether the in vivo administration of imipramine to block MV generation will have similar effects to those observed in our in vitro studies remained unknown. Therefore, we further investigated the in vivo effects of imipramine on bone metabolism using estrogen deficiency-induced bone loss in an animal model.

**Figure 6:**
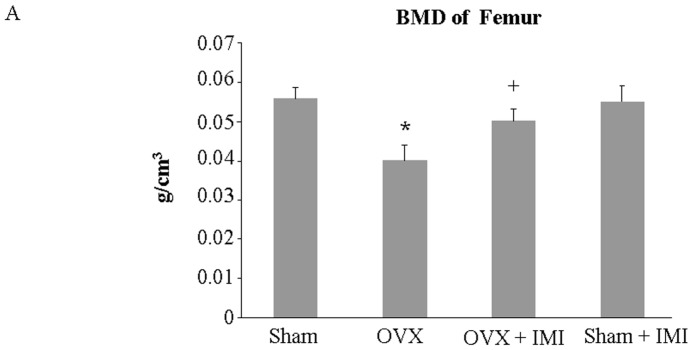

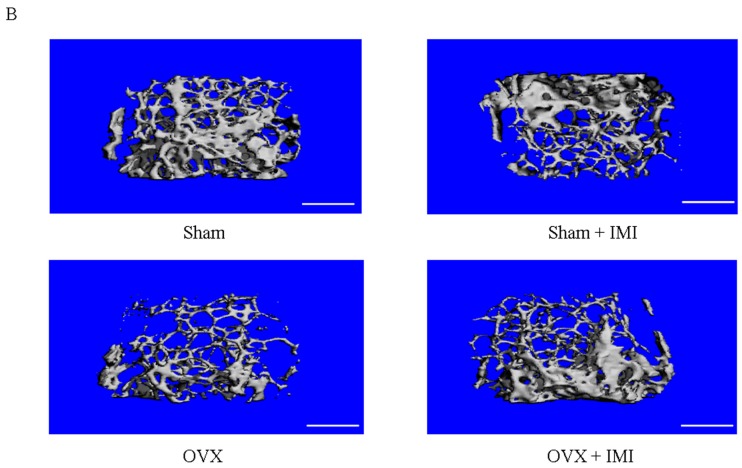
Imipramine protects against ovariectomy-induced bone loss. (**A**) Bone mineral density (BMD) of the femur was assessed on day 3 before mice were sacrificed at the end of the experiment; (**B**) Representative three-dimensional images of spinal bone were generated from micro-CT analysis. Each bar represents the mean ± standard deviation of eight mice. Scale bar, 0.5 mm. Statistical significance determined by two-way ANOVA is designated as follows: * *p* < 0.05 versus sham group; + *p* < 0.05 versus OVX group.

Bone densitometry of the femur showed that administration with imipramine via subcutaneous injection once every other day for 45 days markedly prevented bone mineral density (BMD) decrease in ovariectomized (OVX) mice (Figure 6A). Micro-CT analysis confirmed that bone density was not decreased in imipramine-treated OVX mice (Figure 6B).

**Figure 7:**
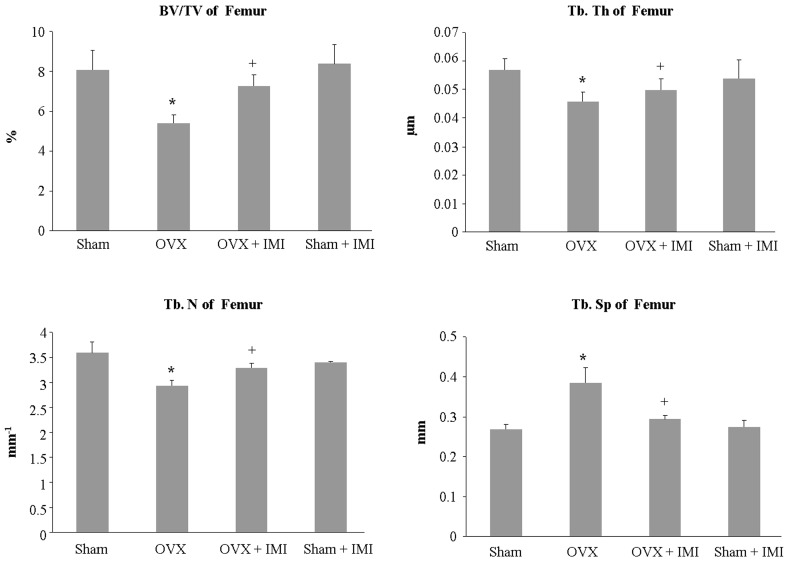
Micro-CT analysis of bone structural parameters. Bone volume (BV/TV, bone volume over tissue volume), trabecular number (Tb.N), trabecular thickness (Th.Th), and trabecular separation (Tb.Sp) were evaluated. Each bar represents the mean ± standard deviation of eight mice. Statistical significance determined by two-way ANOVA is designated as follows: * *p* < 0.05 versussham group; + *p* < 0.05 versus OVX group.

Bone structural parameters were carried out by micro-CT. Quantitative analyses showed that the bone volume (BV/TV), trabecular number (Tb.N) and trabecular thickness (Th.Th) in OVX mice were significantly reduced and trabecular separation (Tb.Sp) was increased compared to the sham group. In contract, imipramine treatment in OVX mice showed an increase in BV/TV, Tb.Th and Tb.N, but a reduction in Tb.Sp (Figure 7).

**Figure 8:**
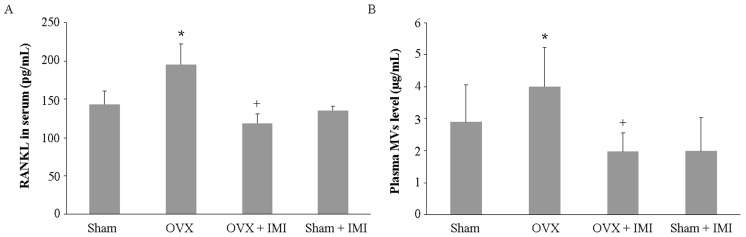
(**A**) Imipramine reduced serum levels of RANKL.Serum RANKL levels were measured by the ELISA method. Each bar represents the means ± standard deviation of eight mice; (**B**) Imipramine reduced plasma levels of MVs. Plasma levels of MVs were determined by measuring MV-associated proteins and are presented as micrograms of MV per milliliter of plasma. Statistical significance determined by two-way ANOVA is designated as follows: * *p* < 0.05 versus sham group; + *p* < 0.05 versus OVX group.

The serum level of RANKLwas evaluated. As shown in Figure 8A, the serum level of RANKL was decreased inimipramine-treated OVX mice. Further experiments on the MV level in plasma showed that the administration of imipramine blocks MV generation in vivo (Figure 8B). All of these results suggested that imipramine indeed protects against bone loss in OVX mice.

## 3. Discussion

Cell–cell communication is a fundamental cellular process and plays an important role in development, tissue homeostasis and pathogenesis of diseases. The maintenance of bone homeostasis mostly relys on cellular communication between osteoblasts and osteoclasts. The receptor activator of nuclear factor-κB ligand (RANKL) is a homotrimeric protein typically membrane-bound to osteoblastic cells, and it exists as a membrane-associated factor that binds to RANK, which is on the surface of osteoclast precursors as a receptor for RANKL, and such recognition induces the differentiation of the precursors into osteoclasts [18]. RANKL/RANK signaling regulates osteoclast formation, activation and survival in normal bone modeling and remodeling and in a variety of pathologic conditions characterized by increased bone turnover. In this process, cell-to-cell contact (osteoblasts to osteoclast precursors) is required for RANKL/RANK signaling.

It is now recognized that microvesicles (MVs) are an integral part of the intercellular microenvironment and may act as regulators of cell–cell communication. MVs may affect target cells by receptor-mediated interactions, leading to target cell stimulation directly or by transferring surface receptors. Once internalized, MVs can fuse their membranes with the plasma membrane of the target cell, thereby resulting in the stimulation of cell signaling or the transfer of various bioactive molecules. For example, in our previous study, osteoblast-derived MVs contained RANKL protein and interacted with osteoclast precursors that were specifically recognized through the receptor ligand (RANKL–RANK), resulting in the stimulation of RANKL–RANK signaling to facilitate osteoclast formation [14].

Microvesicles represent a heterogeneous population of vesicles that range in size from 100 to 1000 nm, which are budded directly from the plasma membrane and contain cytoskeleton and endoplasmic reticulum (ER) elements. They are enriched in some lipids, such as cholesterol, and have high levels of phosphatidylserine (PS) exposed to the outer membrane. Phosphatidylserine is relocated to the outer membrane leaflet, specifically at sites on the cell surface where MV shedding occurs, while the topology of membrane proteins remains intact [19,20]. The mechanism of vesiculation is related to rearrangements in the symmetry of membrane phospholipids. Sphingomyelin (SM), a phospholipid enriched in the external leaflet, is the active component responsible for proangiogenic potential by stimulating endothelial cell migration, invasion, and tube formation [19]. Sphingomyelinase (SMase), an enzyme that catalyzes the hydrolysis of SM to phosphorylcholine and ceramide, is related to MV release. The rearrangements in the symmetry of membrane phospholipids by sphingomyelinase create ceramide gradients across the plasma membrane, leading to the budding of vesicles towards sphingomyelinase activity [21]. Such perturbations result in changes in the phospholipid bilayer organization and physical ‘bend’, exposure of PS on the cell surface, followed by localized changes in the structure of the cellular membrane and cytoskeleton, and the budding and formation of MVs.

Imipramine, the prototypical tricyclic antidepressant (TCA), belongs to the dibenzazepine group and has been used in clinical applications to treat a number of disorders, in particular major depression and neuropathic pain [22]. In the 1970s, the ability of TCA to inhibit acid sphingomyelinase (ASM) by mediating the degradation of ASM was discovered [23]. Recently, an elegant study demonstrated that imipramine inhibited MV generation in vitro [13]. However, direct evidence for the effect of imipramine on bone metabolism in vivo is presently rather scarce. Low bone mineral density is prevalent in patients, mostly in women, with major depressive disorder [24]. Epidemiological studies in large populations of antidepressant users showed that TCA reduced bone mineral density and increased the risk of osteoporosis in older adults [25,26]. Other studies have also indicated that the average rates of hip bone loss among TCA users and antidepressant nonusers were similar in age- and multi-variable-adjusted analyses, and that imipramine treatment does not reduce bone strength [27,28]. The reason for bone loss in patients with major depression is complicated, because this diagnosis is often accompanied with a number of hormonal alterations, including these factors to regulate bone metabolism and homeostasis [29]. Chronic inflammation is a strong trigger of bone loss, and depressed patients are also in a mild pro-inflammatory state [30,31].

Extracellular nucleotides act as signaling molecules to regulate bone cell function through P2X ligand-gated ion channels and P2Y receptors [32]. P2X7R is one of members of the P2X receptor subfamily and is activated by extracellular ATP [33]. P2X7R is expressed in both osteoblasts and osteoclasts. Activation of P2X7R promotes mononuclear cell fusion to form multinucleated osteoclasts [34]. R2XPR signaling up-regulates RANKL expression, induces osteoblasts apoptosis and inhibits bone mineralization [35,36]. P2X7R signaling is also involved in ATP-induced MV generation, and specific P2X7R inhibitors reduce MV shedding from immune cells and non-immune cells [37]. Stress and inflammation enhance extracellular nucleotides release, while imipramine diminishes stress-induced inflammation [38].

In this study, we found that imipramine slightly inhibited the expression of osteoblastic differentiation genes but did not affect PTH regulation for these genes; also, imipramine did not block RANKL-mediated osteoclast formation from osteoclast precursors. However, imipramine significantly blocked MVs released from osteoblasts, resulting in a significant reduction in osteoclast formation when co-cultured with osteoclast precursors with MVs collected from imipramine-treated osteoblasts. This property of imipramine inhibiting MV generation may be particularly important for preventing bone loss caused by estrogen deficiency or by long-term depressants in elderly patients, because imipramine facilitates the reduction of local and circulating RANKL levels, which is the critical factor for osteoclast differentiation, mature and survival.

Bone homeostasis is controlled by a social network of communicating cells that differ in origin and function [39]. MVs are important and underappreciated mediators of cell–cell communication. Until now, there has been no published data to explain how MVs may affect bone homeostasis in vivo, and our results presented here show that imipramine may protect against ovariectomy-induced bone loss, possibly by blocking the production of MVs containing RANKL. Future studies should be performed in other animal models of osteopenia and osteoporosis to confirm these findings.

## 4. Materials and Methods

### 4.1. MV Isolation from Osteoblastic Cell Culture and Plasma

The murine stromal/osteoblastic cell line UAMS-32P (a kind gift from O’Brien, CA, USA) [40] was used for MV production. Cells were cultured in α-minimum essential medium (α-MEM, Invitrogen, Carlsbad, CA, USA) containing 10% fetal bovine serum (FBS, HyClone, Logan, UT, USA). FBS was centrifuged at 16,000× *g* for 1 h at 4 °C and subsequently filtered (0.22 μm) to exclude MVs. Cells were treated by PTH (0.1 µmol/L, Sigma-Aldrich, St. Louis, MO, USA) with or without imipramine (10 µmol/L, Sigma-Aldrich, St. Louis, MO, USA) for 12 h. Culture mediums from about 4 × 10^7^ cells were harvested and MVs were purified by differential centrifugation as described previously [14]. The plasma samples were centrifuged at 2500× *g* for 30 min to remove platelets and cellular debris. Then, MVs were isolated by centrifugation at 16,000× *g* for 80 min at 4 °C. After being washed in phosphate-buffered saline (PBS), MVs were resuspended in PBS and stored at 4 °C.

### 4.2. MV Characterization and Quantification

MVs number and size distribution were determined by the Nanosight LM10 nanoparticle characterization system (Nanosight, Salisbury, UK). The amount of collected MVs was estimated by measuring MV-associated proteins, using a Pierce^®^ BCA Protein Assay Kit (Thermo Scientific™, Shanghai, China).

### 4.3. Osteoclast Formation Assay

MVs released from about 4 × 10^7^ PTH-treated UAMS-32P cells in the absence (control group) or presence of imipramine (IMI group) were concentrated and suspended in 100 μL serum-free α-MEM medium. Then, 10 μL of MV suspension from the control group or IMI group, or 30 μL MVs from the IMI group (IMI-equivalent group) were co-cultured with RAW264.7 cells (3 × 10^5^ cells/well) in 24-well plates (1 mL/well) for 6 days in α-MEM complete medium. On day 3, one-half of the medium was replaced with fresh medium and supplemented with 10 or 30 μL of MVs.

Non-adherent bone marrow cells (2 × 10^4^ cells/well), which were prepared according to the method described by O’Brien CA [41], were cultured in 24-well plates in the presence of 100 ng/mL RANKL plus 10 ng/mL M-CSF for 6 days, or co-cultured with UAMS-32P cells in the presence of 0.1 µmol/L PTH for 6 days. Imipramine (10 µmol/L) was added to the culture medium at the beginning of the cell culture, or 2 or 4 days after the cell culture. On day 3, one-half of the medium was replaced with fresh medium and supplemented with osteoclast differentiation factors or imipramine. After 6 days, cells were fixed with a fixation solution (3.7% formalin in PBS) and stained for tartrate-resistant acid phosphatase (TRAP) by using the aTRACP& ALP double-stain Kit (TaKaRa, Beijing, China). TRAP-positive cells containing more than three nuclei were counted and defined as osteoclasts.

### 4.4. Animals

All animal experiments were approved by the Institutional Animal Care and Use Committees (IACUC) of Jiangsu Institute of Nuclear Medicine (JSINM2010007). C57BL/6 female mice (10 weeks old, about 23–25 g) were purchased from Shanghai Laboratory Animals Center (SLAC; Shanghai, China). They were randomly assigned to several groups with eight mice for each group. They were housed under standardized conditions and given free access to diet and water. After adaptationfor one week, mice were sham-operated or ovariectomized (OVX) bilaterally after being anesthetized by intraperitoneal injection of ketamine/xylazine (5/10 mg/kg body weight). One week after operations, imipramine (10 mg/kg body weight) or vehicle (PBS) was subcutaneously administrated once every other day for 45 days.

### 4.5. Bone Histomorphometric and Micro-CT Analysis

Bone mineral density (BMD) of the left femur was evaluated by dual energy X-ray absorptometry (DXS 4000 Pro system, Carestream, New York City, NY, USA) in vivo. For bone structural determination by µCT analysis, the intact right femur and partial spine (lumbar vertebra 1–4) from each mouse was scanned by using a high-resolution micro-CT SkyScan 1176 (SkyScan, Kontich, Belgium), with a voxel size of 9 µm^3^. The applied X-ray voltage was 50 kV and the X-ray intensity was 167 μA. After scanning, 3D microstructural image data was reconstructed using the SkyscanNRecon software. Structural indices were calculated using the Skyscan CT Analyzer software. According to the guidelines for the assessment of bone microstructure in rodents using micro-CT [42], various bone structural parameters including bone volume (BV/TV, bone volume over tissue volume), trabecular number (Tb.N), trabecular thickness (Th.Th), and trabecular separation (Tb.Sp) were evaluated on day 3 before mice were sacrificed.

### 4.6. Serum Biochemical Marker of Bone Metabolism

Blood samples were collected by retro-orbital puncture at the end of the treatment period prior to sacrifice. Serum was extracted immediately after centrifugation at 2000× *g* for 10 min at 4 °C and was stored at −80 °C until required for bone metabolic marker assays. Serum RANKL levels were determined using mouse ELISA kits according to the manufacturer’s instructions (R&D systems Inc., Minneapolis, MN, USA).

### 4.7. Gene Expression Analysis

To explore the effect of imipramine on bone metabolism in vitro, we determined bone metabolism-related gene expression in UAMS-32P cells. UAMS-32P cells were respectively treated for 12 h with vehicle (PBS), imipramine (1or 10 µM), PTH alone (0.1 µmol/L), or imipramine (1or 10 µM) pre-treated for 1 h and then PTH added for 12 h, or PTH pre-treated for 1 h and then imipramine (1 or 10 µM) added for 12 h. Gene expression was measured by real-time PCR. Total RNA was purified by using TRIzol reagent (Invitrogen, Carlsbad, CA, USA) according to the manufacturer’s instructions. *RANKL*, *OPG*, *BMP2*, *Runx2*, *Osterix*, *SphK1*, *SphK2* and *ribosomal protein S2* (as the housekeeping gene) were amplified from the first-stand cDNA by using Taqman quantitative PCR master Mix (Applied Biosystems, Foster City, CA, USA). The TaqMan assay numbers of the primer/probe sets used are: *RANKL*, Mm00441908_m1; *OPG*, Mm00435452_m1; *BMP2*, Mm01340178_m1; *Runx2*, Mm00501580_ml; *Osterix*, Mm04209856_m1; *SphK1*, Mm00448841_m1; *SphK2*, Mm00445021_m1; *ribosomal protein S2* (forward, 5′-CCCAGGATGGCGACGAT-3′; reverse, 5′-CCGAATGCTGTAATGGCGTAT-3′; probe, FAM–5′-TCCAGAGCAGGATCC-3′–NFQ). PCR amplification and detection were performed on an ABI Prism 7500 Sequence Detection System (Applied Biosystems) according to the manufacturer’s instructions. Relative mRNA levels were calculated using the Δ*C*_t_ method [43]. All mRNA levels are normalized to the housekeeping gene mRNA levels.

### 4.8. Statistical Analysis

Data are presented as the group means±standard deviation. A statistical analysis was performed by using two-way ANOVA with Tukey’s multiple comparisons post hoc test or Student’s *t*-test for two independent samples when appropriate. All data were analyzed using SPSS 19.0 (SPSS Statistics, Inc., Chicago, IL, USA).

## Supplementary Materials

Supplementary materials can be found at www.mdpi.com/1422-0067/18/5/1013/s1.
