# Supplementary material for: Imipramine Protects against Bone Loss by Inhibition of Osteoblast-Derived Microvesicles
Source: Int J Mol Sci. 2017 May 8;18(5):1013. doi: 10.3390/ijms18051013 (PMC5454926; doi:10.3390/ijms18051013)
Supplement: Supplementary file 1 [file ijms-18-01013-s001.pdf]

Supplementary Materials: Imipramine Protects against Bone Loss by Inhibition of Osteoblast-Derived Microvesicles

Lili Deng, Ying Peng, Yuhai Jiang, Yu Wu, Yuedi Ding, Yaping Wang, Dong Xu and Qiang Fu

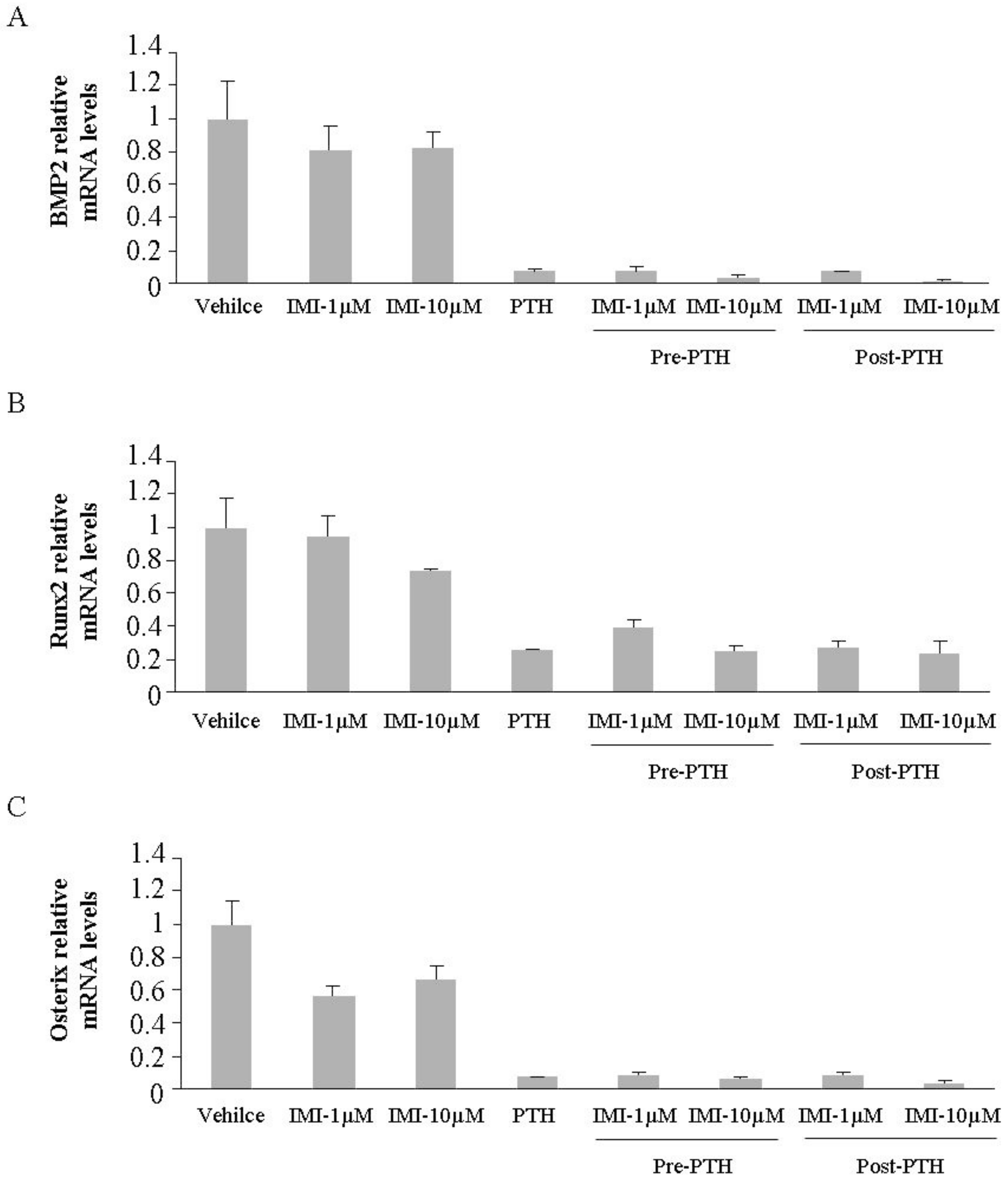

Figure S1. Imipramine regulates genes expression in osteoblast. (**A**) mRNA expression for BMP2; (**B**) mRNA expression for Runx2; (**C**) mRNA expression for Osterix. Each bar represents the mean ± STDEV (n = 3). Vehicle: without any treatments; IMI: with imipramine treatment; PTH: with PTH treatment; Pre-PTH: imipramine pre-treated one hour and then PTH treatment; Post-PTH: PTH pre-treated one hour and then imipramine treatment.
